# Adaptation of Piglets Using Different Methods of Stress Prevention

**DOI:** 10.3390/ani5020349

**Published:** 2015-05-13

**Authors:** Vitaly Bekenev, Arlene Garcia, Vyacheslav Hasnulin

**Affiliations:** 1 Federal State Scientific Institution, Siberian Research and Technological Institute of Animal Husbandry, Novosibirsk reg., Krasnoobsk 630501, Russia; 2 Department of Animal and Food Sciences, Texas Tech University, Lubbock, TX 79409, USA; E-Mail: arlene.garcia@ttu.edu; 3 Federal State Organization, Research Institute of Internal Medicine and Preventive Medicine, Novosibirsk 630089, Russia; E-Mail: hasnulin@ngs.ru

**Keywords:** weanling pigs, stress, food additives, antioxidant activity, lipid peroxidation

## Abstract

**Simple Summary:**

Stressful events play a major negative role in the modern technology of weaned piglets. These events include but are not limited to weaning itself, lack of maternal milk, loss of maternal bonding, mixing of different litters, transportation to growing-finishing farms, and housing conditions. Various additives (phenazepam, aminazine, vitamins E and C, the extract Eleutherococcus senticosus, and ultraviolet irradiation) at different doses and combinations with or without ultraviolet irradiation were used to evaluate their effect on the viability and growth rate of piglets after weaning. Content of lipids in the blood and liver, antioxidant activity (AOA) and lipid peroxidation (LPO) significantly decreased or increased with the use of the additives. Feeding a mixture of additives increased survival rate, average daily gain, and live weight at the end of the experiment.

**Abstract:**

The purpose of this study was to evaluate the viability and growth rate of piglets after weaning, the content of lipids in the blood and liver, antioxidant activity (AOA) and lipid peroxidation (LPO) when various additives are used in feed. The experiments were performed on two crosses of piglets obtained from Large White breed sows and Landrace breed boars. Twenty to 28 animals were randomly assigned per group. The following additives were tested: the benzodiazepine phenazepam, the neuroleptic aminazine, vitamins E and C, and the extract Eleutherococcus senticosus (Araliaceae). Different doses and combinations of the additives against ultraviolet irradiation were used. The addition of these substances improved the growth rate and viability of piglets. AOA increased under the influence of all factors studied, especially with the addition of extract of Eleutherococcus in feed in combination with aminazine and UV-irradiation (*p* < 0.01). However, the addition of Eleutherococcus extract and aminazine intensified LPO (*p* < 0.01), but use of UV irradiation helped to decrease LPO values (*p* < 0.01). Feeding a mixture of additives per pig per day of 3 mL of Eleutherococcus extract, 80 mg of 25% tocopherol, and 500 mg of ascorbic acid increased survival rate, average daily gain, and live weight at the end of the experiment. Thus, the use of prophylactic antistress and sedative drugs during weaning helps AOA normalize LPO of red blood cells; enhance post weaning growth of the pigs by 4.8% to 24.6% and increases piglet survival rate by 5% to 5.1%.

## 1. Introduction

Stressful events play a major negative role in the modern technology of pig-breeding, which results in impairment of survival, livestock yield, reproductive function and the quality of production. The causes of stress for weanling pigs are weaning itself, mixing of different litters, transportation to another area, housing conditions, and nourishment. Different drugs can be used with the purpose of increasing piglet viability and growth rate [1,2,3]. The use of ascorbic acid can regulate synthesis of adaptive substances. Antioxidants can promote the saving of energetic substances and coferments. Vitamin E can reduce strain during stress, and others, such as aminazine can block stress reactions. Anti-stress premixes, including various vitamins, antibacterial agents and plant extracts are widely used [4,5,6,7,8]. Some new drugs such as phenazepam, a benzodiazepine with tranquilizing, soporific, and anticonvulsive properties can also be used [9]. Phenazepam is also known to remove vegetative abnormalities during emotional tension and stress and decreases skeletal musculature tonus. Compared to other drugs, such as diazepam (seduxen), chlordiazepoxide (elenium), nitrazepam (eunoctin), phenazepam exhibits a more pronounced tranquilizing action. The use of extract from the roots of Eleutherococcus senticosus is of great interest. Eleutherococcus senticosus is similar to Siberian Ginseng but it is a different botanical species and has different biological effects. The active agents of this extract are glycosides: А, В, С, D, Е and F. Just as ginseng, Eleutherococcus has an increased antioxidative activity and is capable of improving overall non-specific body resistance [10]. Biological reserve of the air-dry substance of Eleutherococcus senticosus in Russia is about 85 000 tons. Our goal was to study the effect of supplementary feeds: vitamin E, ascorbic acid, aminazine, phenazepam, and a promising, yet insufficiently studied adaptogen, Eleutherococcus extract (including its use on the background of UV irradiation)—on vitality, growth rate, antioxidative activity (AOA) and lipid peroxidation (LPO) in the blood and liver of piglets after ablactation.

## 2. Experimental Section

The experiments were performed on piglets obtained from the crossing of Large White breed sows and Landrace breed boars. In each experiment (except for experiment 1) 25 to 28 piglets were randomly placed into groups according to their age, size, and weight. The piglets’ feed was enriched with various additives, 5 days before weaning (22–26 days of age) and 5–10 days post weaning (31–36 days of age). The experiments were conducted until the piglets reached 12 to 15 weeks of age.

In the first experiment, the anti-stress drug phenazepam was used. Piglets were weaned at 20 days of age and were placed in three groups of 20 piglets. On the first day of the experiment, 3 mL of 0.002% oil emulsion of phenazepam was added to feed for animals in group I, on the second day it was reduced to 2 mL and on the third day it was reduced to 1.5 mL. The piglets in the group II did not receive the drug. However, to soften post weaning stress, 5 days prior to weaning the three litters that made up group II had access to the adjacent farrowing stalls through open passageways. The passageways allowed group II to communicate with the other litters of pigs. Group III was used as the control group (Table 1).

**Table 1:** Least squares means ± SEM for influence of phenazepam or passageways in Experiment 1. Weanling pig body weights and survival rate varied by group and age. N = 20 weanling pigs per group.

Group	BW of a piglet (kg) at the age of (days)				Survival (percent)
	20 d	51 d	74 d	98 d	
phenazepam	5.5 ± 0.13	11.2 ± 0.27 ***	18.3 ± 0.45 *	32.5	100
passage-ways	5.4 ± 0.17	10.6 ± 0.45 *	17.9 ± 0.80	31.5	95
control group	5.6 ± 0.27	9.4 ± 0.43	15.5 ± 1.02	28.3	95

*: Difference from control is significant at *p* < 0.05; ***: Difference from control is significant at *p* < 0.001.

In the second experiment, the piglets, in addition to their basic diet, received the following: group I received 20 mg of vitamin E; group II received 60 mg of vitamin E; group III received 3 mL of eleutherococcus extract; group IV received 6 mL of eleutherococcus extract; and group V was used as the control group.

In the third experiment group I received 6 mL of eleutherococcus extract, group II received 18 to 22 mg of aminazine, group III received 6 mL of eleuthoercoccus + 18 to 22 mg of aminazine and were additionally exposed to UV radiation with an LE-30-1 lamp at a dose of 80 to 90 mer’h/m2, group IV was also exposed to the same dose of UV irradiation, yet without drug additives to the feed ration, and group V was used as the control group.

In the fourth experiment, eleutherococcus extract, vitamin E and C were added to the feed without preliminary mixing. Group I was given 6 mL of eleutherococcus extract + 20 mg of vitamin E, group II was given 3 mL of eleutherococcus extract + 20 mg of vitamin E + 500 mg of ascorbic acid, group III was given 6 mL of eleuthercoccus extract, and group IV was the control. Vitamin E and C were diluted with small amounts of water and shaken to form an emulsion that was fed to the piglets before feeding. The daily dose of additives was given to each group in the morning.

Blood samples from six piglets from each group were analyzed. The blood samples were collected from the lateral auricular vein of each 36-day old piglet. In the third experiment, three piglets from each group were euthanized and the livers of the piglets were examined (Table 2).

**Table 2:** Least squares means ± SEM for growth rate and vitality of weanling pigs under the influence of anti-stress additives. Weanling pig body weights, average daily gain (ADG), and survival rate varied by experiment and group.

Group	No. of animals	BW(kg) in the beginning of experiment	BW (kg) at the end of experiment	ADG (g)	Survival (percent)
**Exp. 2**					
I—vitamin E, 20 mg	28	6.2 ± 0.19	35.3 ± 0.89 *	364	92.8
II—vitamin E, 60 mg	28	6.4 ± 0.17	32.6 ± 1.13	327	85.7
III—eleutherococcus, 3 mL	28	6.3 ± 0.28	34.7 ± 1.17	355	85.7
IV—eleutherococcus, 6 mL	28	6.3 ± 0.12	34.1 ± 1.15	347	100
V—control group	28	6.3 ± 0.13	32.6 ± 0.77	331	89.3
**Exp. 3**					
I—eleutherococcus, 6 mL	25	6.0 ± 0.10	31.6 ± 0.53 ***	320	100
II—aminazine, 20 mg	25	6.1 ± 0.11	31.9 ± 0.65 ***	322	100
III—eleutherococcus, 6 mL + aminazine, 20 mg + UV irradiation	25	6.0 ± 0.14	31.4 ± 0.56 ***	317	91
IV—UV irradiation	25	6.0 ± 0.09	30.8 ± 0.55 **	310	86
V—control group	25	6.0 ± 0.12	27.9 ± 0.54	274	100
**Exp. 4**					
I—eleutherococcus, 6 mL + vitamin E, 20 mg	26	5.7 ± 0.10	36.1 ± 0.11 ***	395	100
II—eleutherococcus, 3 mL+ vitamin E, 20 mg + vitamin C, 500 mg	26	5.7 ± 0.11	37.5 ± 0.56 ***	412	100
III—eleutherococcus, 6 mL	26	5.7 ± 0.10	34.6 ± 0.57 *	375	88.5
IV—control group	26	5.7 ± 0.08	32.6 ± 0.80	349	96.1

*: Difference from control is significant at *p* < 0.05; **: Difference from control is significant at *p* < 0.01; ***: Difference from control is significant at *p* < 0.001.

Lipid peroxidation (LPO), total lipids, total and free cholesterol, vitamin E (in the blood samples as well as in the liver samples) were measured. Antioxidative activity (AOA) in lipids of erythrocytes and livers were estimated by inhibition of thermal oxidation of methyl oleate in the presence of extracted lipids according to previous reports [11]. LPO was measured by the change in conjugated diene content [12]. Total cholesterol and free cholesterol content, as well as vitamin E in the blood serum were also measured according to previous reports [13,14].

### Statistical Analysis

The data were analyzed in Statistics 6.1 for Windows. Results are presented as Least squares means ± standard error of the mean (SEM). The statistical model included the effects of treatment, pen within treatment, time, and all possible interactions. Pen within treatment was used as the model’s error term. All data were tested for outliers using Tukey’s procedure. An F-protected Least Significant Difference test was also used.

## 3. Results and Discussion

### 3.1. Experiment 1

Phenazepam, as well as diazepam have shown to reduce stress reactions, and have a positive effect on the growth and vitality of piglets [15]. At 51 days of age, the piglets' live weights in group I were 1.81 kg greater than the live weight of the piglets’ in the control group (*p* < 0.01) (Table 1). The live weights of group II were 1.26 kg greater than that of the control group (*p* < 0.05). The difference between groups I and II was not significant (*p* > 0.05). At 74 days of age the piglets’ live weights in group I were 2.83 kg greater than the live weight of the piglets’ in the control group (*p* < 0.05). The differences in weight among groups I and II were respectively, 0.55 kg at 51 d, 0.39 kg at 74 d, and 1 kg at 98 d. Thus, there was not a significant difference in live weights between piglets that were given phenazepam and those who had access to passageways (Table 1). Survival rate for piglets in group I was 100% compared to 95% in groups II and III. Average daily gain (ADG) was 347 g in group I, 334 g in group II, and 291 g in the control group. Thus, addition of phenazepam increased live weight by 19.2%, while the use of passageways increased it by 14.8% compared to the control group.

### 3.2. Experiment 2

Under the influence of vitamin E (20 mg dose) piglets in group I had a significant increase in body weight compared to the control group (*p* < 0.05; Table 2; Exp. 2). Although, addition of eleutherococcus did aid in increasing body weight and ADG, its effects were not significant (*p* > 0.05).

The serum levels of vitamin E were 4.20 µg/mL for group I (*p* < 0.05), 5.82 µg/mL (*p* < 0.1) for group II, 5.29 µg/mL (*p* < 0.05) for group III, 3.11µg/mL (*p* < 0.05) for group IV, and 1.69 µg/mL for the control group. It should be noted that the concentration of vitamin E in the mixed feed consumed by piglets was 58 mg/kg which is close to the technology-based standard. The piglets in groups III and IV who received eleutherococcus with feed, exceeded the control animals in the concentration of total lipids in blood serum, total protein, albumin fraction, nucleic acids, and a tendency of superiority in gamma globulins. The piglets that received different doses of vitamin E (groups I and II) did not differ in hematological indexes.

### 3.3. Experiment 3

The use of eleutherococcus at 6 mL (Group I), 20 mg aminazine (Group II), and elutherococcus at 6 mL + 20 aminiazine + UV irradiation (Group III) caused significant increase in piglet body weight gain at the end of the experiment compared to the control group (*p* < 0.01; Table 2; Exp. 3). The use of UV irradiation (Group IV) on its own also showed to have caused significantly higher body weights in piglets at the end of the experiment compared to the control group (*p* < 0.05). UV irradiation was not applied in full dose because it would have increased stress in addition to the current weaning stress the piglets’ were going through. Groups I and II did not differ in percent survival rate from the control group. However, group III and IV had a lower percent survival rate 9% and 14% lower compared to control group, respectively.

Significant differences in the activity of endogenic antioxidants of lipids of erythrocytic membranes were observed in the different groups of the piglets (Table 3). The blood AOA increased under the influence of all the factors in the study, especially when only adding eleutherococcus to the feed (*p* < 0.01; Table 3; group I), and in combination with aminazine and UV irradiation (*p* < 0.01; Table 3; group III). When adding eleutherococcus and aminazine to the feed LPO increased (*p* < 0.01; Table 3; group I and II), but under exposure to UV irradiation it decreased (*p* < 0.01; Table 3; group IV). The ratio AOA/LPO almost in all experimental groups was higher than in the control group, but was the highest in groups III and IV where UV irradiation was applied (*p* < 0.01). Under the action of eleutherococcus, concentration of total lipids in the erythrocyte membranes increased (*p* < 0.05; Table 3; group I). In piglet livers, variations of AOA, LPO, AOA/LPO ratio and total lipids amounts were noticed. The index AOA/LPO increased in all experimental groups (*p* < 0.01). An increase of total lipid concentrations in the piglet livers’ was only observed with the use of eleutherococcus (*p* > 0.05; Table 3; group I). Aminazine (group II) inhibited stress progression to some extent, this was concluded by the low relative mass of the piglets’ adrenal glands. Aminazine (group II) inhibited stress progression to some extent, this was concluded by the low relative mass of the piglets’ adrenal glands (*p* < 0.05). Eleutherococcus and UV irradiation did not reduce the activity of the adrenal cortex (Table 3; group I, III and IV).

### 3.4. Experiment 4

In the fourth experiment, mixtures and additives to eleutherococcus extract were given. Body weight at the end of the experiment increased significantly for all treatment groups (Table 2; Exp. 4). Percent survival rate for the piglets at growing was 100% with the use of 6 mL of eleutherococcus + and 20 mg of vitamin E (Table 2; group I) and with the use of 3 mL of eleutherococcus + 20 mg of vitamin E, and 500 mg of vitamin C (Table 2; group II), 100% and 96.1% compared to the control group, respectively. The use of 6 mL eleutherococcus (Table 2; group III) decreased the percent survival rate compared to the control group.

Average daily gain with the use of 3 mL of eleutherococcus + 20 mg of vitamin E, and 500 mg of vitamin C (Table 2; group II) was 18% higher than the control group. In addition to the increase in average daily gain, body weight at the end of experiment was 15% higher than that in the control group and made up 4.9 kg. Using 3 mL of eleutherococcus + 20 mg of vitamin E, and 500 mg of vitamin C (Table 2; group II) was more effective in average daily gain by 37 g, or by 10% compared to using 6 mL of eleutherococcus alone.

The use of 3 mL of eleutherococcus + 20 mg of vitamin E, and 500 mg of vitamin C (Table 3; Exp. 4; group II) improved metabolism, significantly increased antioxidative activity of the blood erythrocyte lipids (*p* < 0.01), and seemed to reduce the negative impact of stress factors. In spite of LPO increasing in groups I, II, and III compared to the control group (group IV), prevailing processes of accumulating antioxidants reserves over free-radical reactions were observed. This was indicated by increase of the AOA/LPO index under the influence of the drug complex in groups I, II, and III. All groups improved vital functions of the animals, especially group II (100% survival rate). By 98 days of age, there was a significant increase in live weight in group I by 10.7% (*p* < 0.01) and in group II by 15.0% (*p* < 0.01), compared to the control group. Survival rate of the animals in the control group was 96.1% at that age, 3.9% lower than group I and II, but 7.6% higher than group III (Table 2; group III).

**Table 3:** Least squares means for conditions of the piglets’ lipid metabolism under the influence of vitamins C, E, eleutherococcus extract, UV irradiation and aminazine.

Index	GroupI: eleutherococcus, 6 mL	GroupII: aminazine, 20 mg	GroupIII: eleutherococcus, 6 mL + aminazine, 20 mg + UV irradiation	Group IV: UV irradiation	GroupV: Control
**Exp. 3**					
Blood: AOA, h’mL/g	258.3 ± 18.29 ***	201.7 ± 16.20 ***	276.7 ± 13.30 ***	186.0 ± 8.13 **	85.2 ± 17.91
LPO, conditional units	288 ± 15 ***	208 ± 12 **	173 ± 13	50 ± 3 **	128 ± 22
Index AOA/LPO	0.89 ± 0.015	0.98 ± 0.111	1.60 ± 0.067 ***	3.75 ± 0.358 ***	0.59 ± 0.108
Total lipids, mg%	521.3 ± 32.8*	437.3± 24.1	409.0 ± 29.0	431.6 ± 26.1	405.5 ± 14.1
Liver: AOA, h’mL/g	135.0 ± 14.8 **	158.0 ± 15.8 ***	145.0 ± 15.9**	115.0 ± 5.8 **	65.0 ± 9.6
LPO, conditional units	430 ± 11 **	483 ± 7	443 ± 21 **	385 ± 8 ***	493 ± 7
Index AOA/LPO	0.31 ± 0.020 ***	0.33 ± 0.026 ***	0.33 ± 0.035 **	0.30 ± 0.008 ***	0.13 ± 0.012
Total lipids, mg%	2361 ± 199	1887 ± 151	2012 ± 159	2037 ± 94	2037 ± 80
Weight gain, % relative to control group	116.8	117.5	115.7	113.1	100.0
Relative mass of adrenal glands, %	0.0071	0.0048 *	0.007	0.008	0.0073
Survival, %	100.0	100.0	91.0	86.0	100.0
**Exp. 4**					
Index	Group I: eleutherococcus, 6 mL + vitamin E, 20 mg	Group II: eleutherococcus, 3 mL + vitamin E, 20 mg + vitamin C, 500 mg	Group III: Eleutherococcus 6 mL	Group IV: Control	_
Blood AOA, h’mL/g	156.3 ± 27.5 ***	97.7 ± 16.30 ***	79.0 ± 26.59 *	8.3 ± 8.35	_
LPO, conditional units	280 ± 120	390 ± 70 *	220 ± 220	200 ± 50	_
Index AOA/LPO	0.56 ± 0.035 ***	0.25 ± 0.022 ***	0.36 ± 0.033 ***	0.04 ± 0.011	_
Total lipids, mg%	263.3 ± 26.06	266.7 ± 31.83	285.0 ± 15.04	218.0 ± 30.65	_
Weight gain, % relative to control group	113.2	118.0	107.4	100.0	_
Relative mass of adrenal glands, %	0.006	0.006	0.0061	0.0071	_
Survival, %	100	100	88.5	96.1	_

*: Difference from control is significant at *p* < 0.05; **: Difference from control is significant at *p* < 0.01;***: Difference from control is significant at *p* < 0.001.

The relative mass of adrenal glands in all experimental groups turned out to be lower by 14%–16% than that in the control group, which witnesses to less manifestation of the stress reaction (the adrenal glands mass increasing relates to hypersecretion of corticosteroids). The relative mass of thymus was higher in experimental group II and group IV, 0.22% and 0.17%, respectively. This finding indicates the lower involution of the gland in response to the lower stress reaction. Content of vitamin E in the piglets’ liver turned out to be almost twice as high as that in the control group. The amount of vitamin E increased as well.

Application of the proposed additives intensified the animals’ growth rate not only during the first month, but in the course of 62 days of the following monitoring. Many plant species of families Labiatae, Myrtaceae and others, playing an important role in chemoprophylaxis of diseases related to lipids peroxidation, are recognized as a source of natural antioxidants [4]. It is shown that Echinacea purpurea can be used as an additive to achieve immunostimulating effects and increase of feed conversion in pigbreeding [5]. Adding extract of the plants Carvacrol (from Origanum spp.), 3% Cinnamaldehyde (from Cinnamonum spp.) and 2% Capsicum Oleoresin (from Capsicum annum) to the feed for early-weaning piglets decreased amount of intraepithelial lymphocytes in empty intestine and increased presence of lymphocytes in large intestine [6]. Vegetable extracts of cinnamon, thyme and oregano reduce the spread of colon bacillus [7]. The experience in using phytogenic feed additives, such as extracts of Oregano, Rosemary, Yucca, Coriander, and other additives confirms the assumption that they can be promising in feeding pigs and birds to promote production efficiency and productivity. They can be added to a set of non-antibiotic additives [8]. However, there are few data in the literature on the action of such natural antioxidants as eleutherococcus on stress resistance, vitality, and growth rate of animals, especially with piglets at the moment of weaning.

Eleutherococcus extract enhances protein synthesis in an organism with deficit of it, increases antibody dilution when immunizing, and enhances appetite and growth gain of the animals with growth retardation. It has a positive influence on RNA synthesis and increases muscular efficiency as a result of less carbohydrates expenditure and early lipids mobilization [10]. Eleutherococcus glycosides have a protective action in animals under stress, prevent fast involution of thymus [16], is favorable for less hypertrophy of the adrenal glands and protects the loss of glycogen supply in liver [17]. Eleutherococcus glycosides increase conjugation of oxidation and phosphorylation in an organism under stress which provides an optimal level of organism stability. Perhaps, they have, along with ginseng's glycosides, an increased antioxidative activity. Eleutherococcus has a positive effect on vitaminization, it increases saturation of an organism with vitamins B1 and B12 and acts as a synergist of vitamin PP [18]. Eleutherococcus extract decreases excretion of ascorbic acid from an organism, especially under stress.

It is assumed that eleutherococcus does not block stress reaction. Glycosides neutralize glucose capturing inhibitor that arises in blood during stress. This results in glycolysis intensification, generation of the energy sufficient to increase nonspecific resistance, and activation of the processes of fast mobilization and energy recovering. An interference of adaptive hormones appears to be unnecessary, and the hormone system functions with less strain [10].

During the process of adaptation to inadequate environmental factors antioxidants and lipid peroxidation [19] are a key factor. Pathological changes in organisms during adaptation are believed to be linked with accumulating lipid peroxidation products: free radicals, peroxy radicals, hydroperoxides, aldehydes and ketones. Inhibition and normalization of free-radical oxidation is carried out by antioxidants. These are compounds of various types, including tocopherols, ascorbic acid and others [20,21,22,23].

With an increase of vitamin E content in the pigs ration, tocopherol concentration in the blood plasma and liver increases, and antioxidant status of growing pigs improves [24,25]. Previous findings [2] showed that receiving 40 to 60 IU/kg of the vitamin in the feed with addition of fat led to nearly constant balance of alpha-tocopherol concentrations in blood serum and tissues. Without adding fat, the amount of vitamin E must be kept at levels of 80 to 100 IU/kg for the piglets with live weights of 5 to 20 kg. Adding ascorbic acid to piglets' feed during the first 2 weeks after weaning has been reported to lead to a tendency toward growth rate increase and feed conversion [1]. Adding tocopherol to sow feed, and vitamin C to piglet feed after weaning has led to an increase of tocopherol content in their liver and blood [3]. Maternal supply of tocopherol and vitamin C has been reported to enhance piglet immune status after weaning [3]. Adding vitamin C (300 mg/kg of feed) and E (150 mg/kg of feed) leads to the improvement of lumbar muscle color and meat quality of slaughtered pigs (106.0 ± 8.6 kg of body weight) [26]. The efficiency of combining additives, such as ascorbic acid, alpha-tocopherol and oregano (the latter having high content of antioxidants) in feed rations has been reported to prevent stress is chickens [27].

The current study showed that application of eleutherococcus extract and vitamin E, either separately or in combination, as well as using them together with ascorbic acid as a feed additive, has a positive effect on the level of endogenous antioxidants, total lipids content and their peroxidation in cell membranes of the tissues of weanling pigs. In stress conditions energy demands of an organism rise sharply, and there is an augmentation of incoming carbohydrates, and lipids to the blood, which favors an increase of free radical oxidation. The products of non-enzymatic lipid peroxidation have a harmful effect on the enzymes of tricarboxylic cycle and macroergic ATP compounds [28]. In addition, an anti-stress efficiency of the method proposed by us in the current study, was independently estimated by the the institute of Non-contagious Diseases of Animals as requested by the Russian Ministry of Agriculture. They concluded that only glucose content after feeding the preparations to the piglets was higher in the experimental group. Weaning caused indigestion in some piglets’ during the first 3 to 5 days. In the course of a month 5.7% of piglets in the control group died. There was no mortality in those groups of piglets which received eleutherococcus together with vitamins E and C or eleutherococcus only [29].

It was shown in several studies that increased peroxidation (free radical oxidation) of lipids is necessary for switching metabolism processes to a new higher energy level [30,31]. In that case endogenous antioxidants, acting as "traps" for the products of lipid peroxidation, prevent damaging effects of intense free radical oxidation. Extended amounts of stress exhausts the supply of endogenous antioxidants and, with the negative balance of antioxidants incoming with feed, this leads to lipid peroxidation going out of antioxidant control. Increased lipid peroxidation becomes a damaging agent, rapidly exhausting adaptive reserves of a biosystem. It is very likely that in piglets too, under great stress and shortage of antioxidants supply, an intensification of oxidation chain reactions occur, and processes of supplying energy to the main functions of vital activity may collapse and cause death.

During the first days after weaning, stress is a big factor for piglets, and under the action of freeradical oxidation products [21] live weights decrease, and atonia, anemia, indigestion, water retention and death may occur.

According to the experimental data collected, the additives applied, vitamin E, eleutherococcus, ascorbic acid and aminazine, increase antioxidative activity of lipids. Peroxidation is a regulating factor in the direction of oxidation processes. Prolonged stress exhausts the supply of endogenous antioxidants, and with insufficient intake of antioxidants from feed, lipid peroxidation increases. Because of the advantage of enzymatic oxidation over the free-radicals, high-energy products (fats, carbohydrates) are consumed for their intended purpose, growth and development of the animals, which is favorable for improving adaptive stability and vital activity of piglets.

## 4. Conclusions

Phenazepam, aminazine, and especially Spiny Eleutherococcus, in combination with vitamins E and C, promote an increase of antioxidative activity in the weanling pigs’ tissues, improve their energy status, enhance synthesis processes, improves adaptation, and growth of the piglets subjected to stress factors. As a result, applying prophylactic anti-stress drugs and tranquilizers to the weanling pigs as feed additives makes it possible, to normalize the antioxidative activity and peroxidation of erythrocytic lipids. Additionally, the application of these prophylactic anti-stress drugs and tranquilizers enhances post weaning growth by 4.8% to 24.6% and increase survival rate by 5% to 5.1%. These factors can become accepted production practices for growing weanling pigs.
